# The anti-inflammatory effects of SGLT inhibitors

**DOI:** 10.18632/aging.102175

**Published:** 2019-08-25

**Authors:** Álvaro García-Ropero, Carlos G. Santos-Gallego, Juan J. Badimon

**Affiliations:** 1 Atherothrombosis Research Unit, Mount Sinai Heart, Icahn School of Medicine at Mount Sinai, New York, NY 10029, USA; 2 Cardiology Department, Imperial College London, The Royal Brompton and Harefield Hospital, London, United Kingdom

**Keywords:** diabetes mellitus, cardiovascular mortality, inflammation, SGLT inhibitors, empagliflozin, anti-inflammatory effects

The newest class of antidiabetic drugs, the sodium-glucose cotransporter type 2 inhibitors (SGLT2i) have gained tremendous importance after the surprising results of the EMPA-REG OUTCOME trial published in 2015 by Zinman et al. In this clinical trial, empagliflozin significantly reduced the cardiovascular (CV) death by 38% in patients with type 2 diabetes mellitus (T2DM), compared with placebo. These outstanding cardiac outcomes were mostly achieved by a noteworthy reduction in hospitalization for heart failure (HF). Therefore, the scope of the scientific community has been attracted to elucidate the biochemical and molecular mechanisms responsible for such benefits.

Among all SGLT receptors (SGLT-R), which are involved in facilitating the transport of glucose from the bloodstream into the cardiac cells, subtypes 1 and 2 have been the most widely studied. The former are expressed in the heart (abundantly among HF patients). Contrarily, SGLT2-R are not found in the cardiac cells and they act primarily in the proximal convoluted tubule (PCT) in the kidneys [1]. The absence of SGLT2-R in the heart, along with the fact that antidiabetic drugs will take years to exert CV benefits by lowering glucose levels, makes more challenging to interpret the mechanism of action of SGLTi. Few metabolic theories have been postulated to explain such CV benefits including: 1) phosphorylation of cardiac adenosine monophosphate-activated protein kinase (AMPK), which promotes FA oxidation and thus, greater adenosine triphosphate (ATP) production; and 2) cardiac shift towards ketone bodies (KB) consumption over fatty acids (FA) or glucose, which ultimately would lead to a more oxygen-efficient energy generation [2].

**Figure 1:**
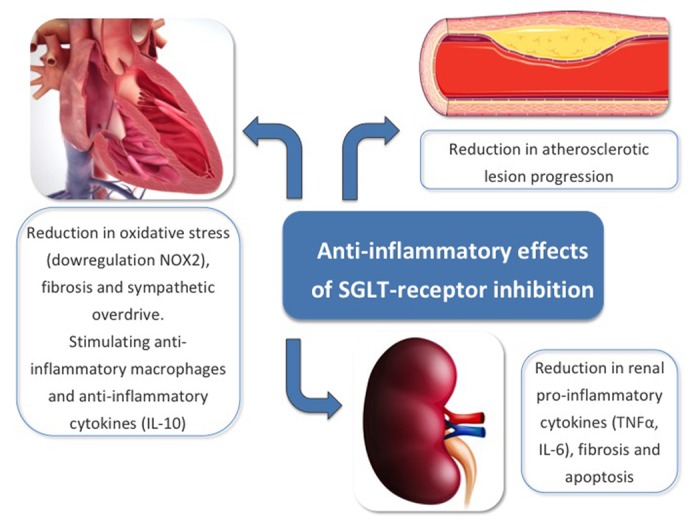
The anti-inflammatory effects of SGLT-R inhibition.

Interestingly, SGLT-R have also demonstrated to play an important role in inflammatory response and thus, this may contribute to mitigate cardiac adverse remodeling and improve CV outcomes (similarly to renin-angiotensin-aldosterone system (RAAS) inhibitors). For instance, SGLT1 knockdown mice model showed less oxidative stress following ischemia reperfusion (I/R) injury and therefore, less myocardial necrosis and infarct size [3]. These effects were mediated via downregulation of NADPH oxidase 2 (NOX2). Likewise, SGLT2i have also been associated with anti-inflammatory effects. Dapagliflozin, significantly reduced collagen synthesis by stimulating anti-inflammatory macrophages and by inhibiting myofibroblast differentiation after myocardial infarction (MI) in rats [4]. In this study, dapagliflozin group also showed greater level of anti-inflammatory cytokine subtype 10 (IL-10). Furthermore, empagliflozin, attenuated activation of human fibroblast activation, via transforming growth factor β1 (TGFβ1), in a dose-dependent fashion [5]. There was also a reduction in pro-fibrotic markers in the empagliflozin group (including collagen type-I α1-chain and matrix metallopeptidase 2, among others). In accordance to this, our group has demonstrated that empagliflozin significantly reduced sympathetic overdrive (i.e. catecholamine levels), which is part of the neurohormonal activation and a major hallmark for myocardial adverse remodeling [6]. Macroscopically, empagliflozin administration for 10 weeks was associated with a reduction in atherosclerotic lesion progression in the aorta of a high-fat diet fed apolipoprotein E (APOE) knockout mice model, with borderline statistical significance (p=0.06) [7]. The authors of this study also demonstrated the expression of SGLT1-R in all aortic samples, whereas SGLT2-R was barely found in only few samples. At the renal level, SGLT2i have also demonstrated to mitigate inflammation. In a diabetes-induced rat model, empagliflozin significantly reduced renal expression of pro-inflammatory cytokines and chemokines (including tumor necrosis alpha (TNFα), urinary markers of kidney inflammation (such as IL-6) as well as apoptosis [8]. In this manuscript, empagliflozin also was associated with less expression of pro-fibrotic genes (i.e. TGFβ, collagen type IV and fibronectin). The anti-inflammatory effects of SGLT-R inhibition are presented on Figure 1.

It has been well established the existence of certain degree of systemic inflammation in patients with DM which is otherwise, partly responsible for the CV complication in such population. This fact may be the reason why SGLTi have showed outstanding benefits in terms of CV mortality among those patients. Nonetheless, these agents also reduced inflammation in non-diabetic models, such us cardiac I/R injury, and that widens the spectrum of patient who may benefit from SGLT-R inhibition, including those with chronic inflammatory disease. Whether inhibition of SGLT-R subtype 1 and/or 2 could potentially add positive results among targeted population needs further investigation.

In conclusion, SGLTi have clearly showed to mitigate cardiac adverse remodeling, improve myocardial function and reduce heart failure mortality beyond glycemic status. Whether this is solely explained by a metabolic substrate shift of cardiac cells an/or whether anti-inflammatory properties of SGLT-R inhibition may also be partly responsible still remains unclear and needs to be addressed in large trials.
